# Estimation of Multiyear Consequences for Abortion Access in Georgia Under a Law Limiting Abortion to Early Pregnancy

**DOI:** 10.1001/jamanetworkopen.2023.1598

**Published:** 2023-03-06

**Authors:** Sara K. Redd, Elizabeth A. Mosley, Suba Narasimhan, Anna Newton-Levinson, Roula AbiSamra, Carrie Cwiak, Kelli Stidham Hall, Sophie A. Hartwig, Johanna Pringle, Whitney S. Rice

**Affiliations:** 1 Center for Reproductive Health Research in the Southeast, Emory University Rollins School of Public Health, Atlanta, Georgia; 2 Center for Innovative Research on Gender Health Equity, University of Pittsburgh School of Medicine, Pittsburgh, Pennsylvania; 3 Amplify Georgia Collaborative, Atlanta; 4 Department of Gynecology and Obstetrics, Emory University School of Medicine, Atlanta, Georgia; 5 Department of Population and Family Health, Columbia University Mailman School of Public Health, New York, New York

## Abstract

**Importance:**

Following the US Supreme Court ruling in *Dobbs v Jackson Women’s Health Organization*, Georgia’s law limiting abortion to early pregnancy, House Bill 481 (HB481), was allowed to go into effect in July 2022.

**Objectives:**

To estimate anticipated multiyear effects of HB481, which prohibits abortions after detection of embryonic cardiac activity, on abortion incidence in Georgia, and to examine inequities by race, age, and socioeconomic status.

**Design, Setting, and Participants:**

This repeated cross-sectional analysis used abortion surveillance data from January 1, 2007, to December 31, 2017, to estimate future effects of HB481 on abortion care in Georgia, with a focus on the 2 most recent years of data (2016 and 2017). Abortion surveillance data were obtained from the 2007-2017 Georgia Department of Public Health’s Induced Termination of Pregnancy files. Linear regression was used to estimate trends in abortions provided at less than 6 weeks’ gestation and at 6 weeks’ gestation or later in Georgia, and χ^2^ analyses were used to compare group differences by race, age, and educational attainment. Data were analyzed from July 26 to September 22, 2022.

**Exposures:**

HB481, Georgia’s law limiting abortion to early pregnancy.

**Main Outcome and Measures:**

Weeks’ gestation at abortion (<6 vs ≥6 weeks).

**Results:**

From January 1, 2007, to December 31, 2017, there were 360 972 reported abortions in Georgia, with an annual mean (SD) of 32 816 (1812) abortions. Estimates from 2016 to 2017 suggest that 3854 abortions in Georgia (11.6%) would likely meet eligibility requirements for abortion care under HB481. Fewer abortions obtained by Black patients (1943 [9.6%] vs 1280 [16.2%] for White patients), patients younger than 20 years (261 [9.1%] vs 168 [15.0%] for those 40 years and older), and patients with fewer years of education (392 [9.2%] with less than a high school diploma and 1065 [9.6%] with a high school diploma vs 2395 [13.5%] for those with some college) would likely meet eligibility requirements under HB481.

**Conclusions and Relevance:**

These findings suggest that Georgia’s law limiting abortion to early pregnancy (HB481) would eliminate access to abortion for nearly 90% of patients in Georgia, and disproportionately harm patients who are Black, younger, and in lower socioeconomic status groups.

## Key Points

**Question:**

What are the anticipated multiyear consequences of Georgia’s law prohibiting abortions after detectable embryonic cardiac activity on abortion care in Georgia?

**Findings:**

In this repeated cross-sectional analysis of 360 972 abortions in Georgia from 2007 to 2017, an estimated 3854 abortions (12%) would likely meet eligibility requirements for abortion care under Georgia’s law. Fewer abortions obtained by Black patients, patients younger than 20 years, and patients with a high school education or less would likely meet eligibility requirements under the law.

**Meaning:**

These findings suggest that Georgia’s law limiting abortion to early pregnancy would eliminate access to abortion for nearly 90% of patients in Georgia and disproportionately harm patients who are Black, younger, and in lower socioeconomic status groups.

## Teaser

This cross-sectional study examines trends in abortion in Georgia from 2007 to 2017 by weeks of gestation and estimates the proportion of abortions that will remain legal in Georgia after the passage of House Bill 481, a restrictive abortion law, goes into effect by patient race, age, and educational level.

## Introduction

Safe, legal, and accessible abortion services remain a cornerstone of comprehensive reproductive health care. The US Supreme Court’s June 24, 2022, ruling in *Dobbs v Jackson Women’s Health Organization* (hereinafter referred to as *Dobbs v Jackson*) ended the federal constitutional protection for abortion.^1^ Many states moved to further criminalize abortion by passing—or petitioning courts to drop injunctions on—hyperrestrictive abortion laws, some of which prohibit abortion early in pregnancy after detection of embryonic cardiac activity (often referred to as “heartbeat bills”^2^ or “6-week abortion bans”). These bans limit abortion to approximately 6 weeks’ gestation—an estimation of when embryonic cardiac activity can typically be detected—often before a person knows they are pregnant.^2,3,4^ In March 2019, the Georgia legislature passed House Bill 481 (HB481), a law prohibiting abortion on detection of embryonic cardiac activity, which was subsequently enjoined via *SisterSong v Kemp*.^2,3^ Following the *Dobbs v Jackson* decision, the 11th Circuit Federal Court of Appeals allowed HB481 to go into effect on July 20, 2022, overruling a lower court’s injunction of the bill.^5^ On November 15, 2022, in response to a second legal challenge to HB481 (via *SisterSong v State of Georgia*),^6^ a Fulton County Superior Court judge ruled HB481 unenforceable, given that the law was unconstitutional when written, passed, and enacted in 2019.^7^ The State subsequently appealed this ruling to the Georgia Supreme Court which, on November 23, 2022, reinstated HB481 pending its review of the case.^8^

Restrictive abortion policies are structural determinants^9^ that dictate who can access health care, and via what means. Laws like HB481 that ban abortion early in pregnancy are a favored political tactic by antiabortion legislators and advocates; these laws fundamentally strip individuals of their reproductive bodily autonomy^10^ and cause harm at any stage in pregnancy.^11^ Major medical groups^12^ oppose such bans, citing their far-reaching consequences for patients and health care practitioners. Numerous studies have documented the negative impacts of gestational limits, including fewer clinics offering abortion care,^13^ increased rates of infant mortality,^14^ decreased accuracy in knowledge of abortion legality,^15^ and overall greater travel distances for abortion.^16^ Additionally, research has documented how gestational limits disproportionately impact patients who are Black. For instance, a previous study on Georgia’s House Bill 954 (HB954), which prohibited abortions after 22 weeks’ from last menstrual period (LMP), found that the law disproportionately affected Black pregnant persons in the state.^17^ We hypothesize that laws such as HB481 will likely reinforce inequities in access to abortion care,^10^ exacerbating barriers for those with comparatively limited access, including Black people, young people, and people with lower socioeconomic status.

Georgia has long served as an abortion access hub in the Southeast.^18,19,20^ House Bill 481, which reduced the legal limit for abortions from 22 weeks to approximately 6 weeks from LMP, severely restricted access to abortion in Georgia and across the region. However, to date, no study has examined the anticipated impact of enactment of HB481 on abortion access in the state. Therefore, we sought to describe trends in abortion by weeks of gestation in Georgia from 2007 to 2017 and estimate the proportion of abortions that will remain legal in Georgia under HB481. Additionally, we sought to examine inequities in these proportions by patient race, age, and educational level, sociodemographic factors reflecting structural inequities in access to comprehensive reproductive health care.

## Methods

### Data Sources

Georgia law^21^ and the Georgia Department of Public Health (DPH) mandate that health care practitioners report each abortion provided in Georgia within 10 days of the abortion through their Induced Termination of Pregnancy (ITOP) surveillance system.^17,22^ We obtained annual, aggregate, cross-sectional ITOP data from DPH via 2 data requests. The first ITOP file contained the total number of abortions in Georgia from 2007 to 2017, and the second contained the number of abortions at less than 13 weeks from LMP in Georgia from 2007 to 2017, disaggregated by week of gestation. Data in both ITOP files were stratified by patient race (data were not disaggregated by ethnicity and race simultaneously), age, and educational level, variables entered by practitioners completing ITOP reporting forms based on patient report. The institutional review board of Emory University reviewed and approved this study, which analyzed deidentified secondary data; no informed consent was necessary. The Georgia DPH granted exempt status for their institutional review board review and approval owing to the use of deidentified public records. This study followed the Strengthening the Reporting of Observational Studies in Epidemiology (STROBE) reporting guideline.

### Measures

Using ITOP data, we report annual data on the total number of abortions provided in Georgia as well as the number and proportions of abortions provided in Georgia by weeks’ gestation, dated using LMP, from January 1, 2007, to December 31, 2017. Since embryonic cardiac activity is typically present by 6 weeks and 0 days’ gestation, we categorized abortions as those likely to be legal in Georgia (<6 weeks 0 days) and those that will likely no longer be legal in Georgia (≥6 weeks 0 days) under HB481. To calculate the number of abortions at less than 6 weeks’ gestation, we summed the number of abortions provided at 1 through 5 weeks’ gestation in each year. Next, to calculate the number of abortions at 6 weeks’ gestation or later, we subtracted the number of abortions at less than 6 weeks from the total number of abortions each year. For years 2016 and 2017—the 2 most recent years of our ITOP data—we assessed differences in abortion gestation date (<6 vs ≥6 weeks) by patient sociodemographic characteristics, including race (Black or White), age (<20, 20-29, 30-39, or ≥40 years), and educational level (less than high school diploma, high school diploma, or some college or higher). Notably, the data were only available in the aggregate and could not be disaggregated by more than 1 sociodemographic variable at once.

### Statistical Analysis

In this repeated cross-sectional analysis, we first present annual data on the total number of abortions and the number and proportion of abortions stratified by weeks’ gestation (<6 and ≥6 weeks) in Georgia from 2007 to 2017 to examine trends in abortion by gestation over time. We used simple weighted linear regression to test annual trends in the total number of abortions provided in Georgia and the number of abortions provided before 6 weeks and at 6 weeks or later in Georgia from 2007 to 2017. Next, we present the numbers and proportions of abortions in Georgia stratified by weeks’ gestation and patient race, age, and educational level from January 1, 2016, to December 31, 2017, the 2 most recent years of data and the calendar years following full implementation of HB954, Georgia’s 22-week gestational limit, in October 2015. Prior research^17,22^ revealed that abortion incidence changed most substantially following full (vs partial) implementation of HB954.^17,22^ Thus, we report results from 2016 to 2017, as these years capture the most recent abortion trends in Georgia. We used χ^2^ analyses to compare the proportion of abortions occurring before 6 weeks and at 6 weeks or later among different racial, age, and educational groups from 2016 to 2017. All years of data for racial, age, and educational group differences are provided in eTables 1 to 3 in Supplement 1. All trends are presented visually in eFigures 1 to 4 in Supplement 1. We conducted 2-sided hypotheses testing using an a priori α of .05. We performed all analyses between July 26 and September 22, 2022, using SAS, version 9.4 (SAS Institute Inc).^23^

## Results

### Abortion Trends in Georgia by Weeks’ Gestation, 2007 to 2017

From January 1, 2007, to December 31, 2017, 360 972 abortions were obtained in Georgia. The annual number of abortions in Georgia fluctuated between 30 000 and 36 000 over the study period, with a mean (SD) of 32 816 (1812) abortions per year (Table 1). In all years of the study period, abortions at 6 weeks’ gestation or later comprised most abortions in Georgia (ranging from 88% to 95% of abortions). Trends in the weeks’ gestation of abortion shifted slightly over the study period, with more abortions occurring earlier in pregnancy in more recent years. The proportion of abortions occurring at less than 6 weeks increased from a low of 5.3% in 2008 to a high of 12.1% in 2017 (β = 178.6 [95% CI, 105.1-252.2]; *P* < .001). Correspondingly, the proportion of abortions occurring at 6 weeks or later declined from a high of 94.7% in 2008 to a low of 87.9% in 2017 (β = −481.9 [95% CI, −826.7 to −137.1]; *P* = .01).

**Table 1.** Number and Percentage of Abortions Provided in Georgia, Stratified by Weeks of Gestation^a^

Year	Abortions by gestational week		Total No. of abortions
	<6 wk	≥6 wk	
2007	2415 (7.2)	31 120 (92.8)	33 535
2008	1926 (5.3)	34 168 (94.7)	36 094
2009	2185 (6.5)	31 244 (93.5)	33 429
2010	2546 (7.3)	32 309 (92.7)	34 855
2011	2702 (8.2)	30 235 (91.8)	32 937
2012	3401 (10.8)	28 234 (89.2)	31 635
2013	3036 (9.8)	27 828 (90.2)	30 864
2014	2522 (8.4)	27 669 (91.6)	30 191
2015	3559 (11.4)	27 541 (88.6)	31 100
2016	3799 (11.1)	30 299 (88.9)	34 098
2017	3909 (12.1)	28 325 (87.9)	32 234
2007-2017, mean (SD) [%]	2909 (673) [8.9]	29 907 (2183) [91.1]	32 816 (1812)
2016-2017, mean (SD) [%]	3854 (78) [11.6]	29 312 (1396) [88.4]	33 166 (1318)

^a^ Data are from Induced Termination of Pregnancy surveillance system, January 1, 2007, through December 31, 2017. Unless otherwise indicated, data are expressed as No. (%) of abortions.

### Abortion Incidence and Inequities in Georgia Under HB481

Based on abortion trend data from 2016 to 2017, the mean (SD) annual number of abortions in Georgia was 33 166 (1318). Of these 33 166 abortions, only a mean (SD) of 3854 (78 [11.6%]) would likely meet the eligibility requirement for legal abortion care in Georgia under HB481 (Table 1). The remaining 29 312 (1396 [88.4%]) abortions would no longer be legal in Georgia under HB481.

When examining differences by patient sociodemographic characteristics, we observed inequities in the proportion of abortions that would likely meet the eligibility requirement for legal abortion care in Georgia under HB481 across racial, age, and educational groups. Compared with White patients, Black patients had a significantly lower proportion of abortions that would likely meet the eligibility requirement for legal abortion care in Georgia under HB481 (Table 2). Using mean data from 2016 to 2017, only 1943 abortions (9.6%) obtained by Black patients would meet the eligibility requirement compared with 1280 (16.2%) obtained by White patients (*P* < .001).

**Table 2.** Number and Percentage of Abortions Provided in Georgia, Stratified by Weeks of Gestation and Patient Race, Age, and Educational Level^a^

Categories by year	Abortions by gestational week, No. (%)		Total No. of abortions	χ^2^_1_ value^b^
	<6 wk	≥6 wk		
**Race**				
2016				
Black	1920 (9.2)	18 944 (90.8)	20 864	256.93
White	1266 (15.8)	6745 (84.2)	8011	
2017				
Black	1966 (10.1)	17 457 (89.9)	19 423	225.62
White	1293 (16.7)	6460 (83.3)	7753	
2016-2017 Mean				
Black	1943 (9.6)	18 201 (90.4)	20 144	241.91
White	1280 (16.2)	6603 (83.8)	7882	
**Age**				
2016				
<20 y	285 (9.4)	2749 (90.6)	3034	28.36
20-29 y	2166 (10.8)	17 935 (89.2)	20 101	
30-39 y	1199 (12.2)	8652 (87.8)	9851	
≥40 y	149 (13.4)	963 (86.6)	1112	
2017				
<20 y	236 (8.8)	2457 (91.2)	2693	63.96
20-29 y	2218 (11.7)	16 670 (88.3)	18 888	
30-39 y	1269 (13.3)	8254 (86.7)	9523	
≥40 y	186 (16.5)	944 (83.5)	1130	
2016-2017 Mean				
<20 y	261 (9.1)	2603 (90.9)	2864	44.29
20-29 y	2192 (11.2)	17 303 (88.8)	19 495	
30-39 y	1234 (12.7)	8453 (87.3)	9687	
≥40 y	168 (15.0)	954 (85.1)	1121	
**Educational level**				
2016				
<HS graduate	412 (9.2)	4069 (90.8)	4481	106.05
HS graduate or GED	1061 (9.3)	10 361 (90.7)	11 422	
Some college or more	2322 (12.8)	15 847 (87.2)	18 169	
2017				
<HS graduate	372 (9.2)	3670 (90.8)	4042	156.57
HS graduate or GED	1068 (9.9)	9761 (90.1)	10 829	
Some college or more	2467 (14.2)	14 867 (85.8)	17 334	
2016-2017 Mean				
<HS graduate	392 (9.2)	3870 (90.8)	4262	130.29
HS graduate or GED	1065 (9.6)	10 061 (90.4)	11 126	
Some college or more	2395 (13.5)	15 357 (86.5)	17 752	

Abbreviations: GED, General Education Development test; HS, high school.

^a^ Data are from Induced Termination of Pregnancy surveillance system, January 1, 2016, through December 31, 2017.

^b^ *P* < .001 for all comparisons within race, age, and educational level groups categories.

Regarding patient age, the youngest patients (aged <20 years) had the lowest proportion of abortions that would likely meet the eligibility requirement for legal abortion care in Georgia under HB481 compared with older patients (aged ≥20 years) (Table 2). Using means from 2016 to 2017, only 261 abortions (9.1%) obtained by those younger than 20 years would meet the eligibility requirement, followed by 2192 (11.2%) obtained by those aged 20 to 29 years, 1234 (12.7%) obtained by those aged 30 to 39 years, and 168 (15.0%) obtained by those 40 years or older (*P* < .001).

Finally, patients with fewer years of education (ie, those with less than a high school education and high school graduates) had a significantly lower proportion of abortions that would likely meet the eligibility requirement for legal abortion care in Georgia under HB481 compared those who completed some college or more (Table 2). Using means from 2016 to 2017, only 392 abortions (9.2%) obtained by patients with less than a high school education and 1065 (9.6%) obtained by high school graduates would meet the eligibility requirement compared with 2395 (13.5%) obtained by those with some college or higher (*P* < .001).

## Discussion

*Dobbs v Jackson* provided a legal precedent for states to enact highly restrictive laws like HB481, which limits abortion to early pregnancy. In this descriptive study, we sought to estimate the proportion of abortions provided in Georgia that would likely be legal under a law like HB481 over multiple years and examine inequities by patient race, age, and educational level. Using a gestational cutoff of 6 weeks and 0 days, we estimate that only 11.6% of abortions provided in Georgia would meet the eligibility requirement for abortion care under HB481; the remaining 88.4% of abortions would no longer be legal. These restrictions will likely result in pregnant individuals seeking self-managed abortion care, traveling out of state to obtain abortion care, or being forced to continue their pregnancy and face the attendant risks of pregnancy-related morbidity and mortality.

Our findings mirror studies of similar restrictions in Texas, where abortions sharply decreased after implementation of a similar law limiting abortion to early pregnancy.^24^ Additionally, early estimates from the Society of Family Planning’s #WeCount project also indicate substantial, though not as drastic, decreases in abortions in Georgia in the early months after HB481 implementation.^25^ These results reiterate that laws limiting abortion to early pregnancy eliminate abortion access and are a threat to personal and public health.^4,26,27^ Most people do not recognize they are pregnant before 6 weeks’ gestation^28^; thus, a law like HB481 creates widespread challenges to accessing abortion and increases the likelihood that people have to carry pregnancies against their wishes.^29^ While Georgia was once a major point of abortion access in the Southeast, people across the region will likely need to travel further—potentially hundreds of miles—to reach care, due to HB481.^30,31^

Our findings also emphasize the inequitable implications of HB481 and suggest that Black people, younger people, and people with fewer years of education will be disproportionately affected by HB481. These findings support and extend the existing evidence on unjust social and health consequences of restrictive abortion policies.^17,32,33,34,35^ In an evaluation of HB954, Black patients accessed abortions later in pregnancy and at higher rates than their White counterparts,^17^ while Roberts et al^18^ reported disproportionate effects of HB954 for those with a high school education or less. Undoubtedly, individuals at the intersection of these identities (eg, young Black people, young people with fewer years of education) are at the highest risk for experiencing compounded disproportionate effects of HB481 by way of multiple, simultaneous forms of oppression via racism and White supremacy, classism, and ageism.

Barriers to abortion care—including poverty, lack of transportation, lack of language equity, medical racism, and immigration enforcement^36^—will be heightened by HB481, particularly for those already experiencing inequitable access to care. Prior to *Dobbs v Jackson*, clients seeking abortion funding assistance from Georgia’s local abortion fund, Access Reproductive Care–Southeast, predominantly identified as non-Hispanic Black, were 18 to 34 years of age, and had a high school education or less.^37^ Therefore, people seeking abortions in the South are often those with the fewest resources and who experience the greatest burden of intersectional and structural obstacles to care.^38^ As people seeking abortion care are increasingly forced to travel out of state, access barriers will compound to further heighten inequities, stratifying the ability to access care between those with advantaged positions in society and those without, thus increasing the risk of health, social, and economic consequences of reduced access to abortion care (eg, adverse birth outcomes and infant and maternal mortality).^14,39,40,41,42,43,44,45,46,47^

Indeed, our findings carry ominous implications for maternal mortality and morbidity in Georgia, a state with one of the highest maternal mortality rates in the nation^48^ and where Black women are over twice as likely to die from pregnancy-related complications compared with White women.^49^ Similarly, a national study simulating changes in maternal mortality resulting from a hypothetical total abortion ban predicted a 21% increase in maternal mortality overall and a 33% increase for Black people.^47^ With HB481’s increased restrictions on abortion in Georgia, these outcomes will likely worsen, particularly given Georgia’s severe obstetric care shortage where roughly half of counties lack an obstetrician.^50^ A recent survey of Georgia residents found that if abortion were to become illegal in Georgia, 94% of residents reported anticipating a range of negative emotional responses, and 8% reported considering self-harm behaviors (eg, intentional abdominal trauma, suicide).^51^ Together, these findings and context encapsulate the significant physical and emotional toll pregnant people face under HB481.

### Limitations

There are several limitations to consider when examining our descriptive study. First, we were unable to obtain more recent years of data (beyond 2017) on abortion incidence in Georgia by weeks’ gestation. Despite this, our data provide some sense of the multiyear effect of HB481 on abortion access in Georgia before the release of ITOP data, often months to years after collection. Second, the operationalization of some ITOP variables—including estimated weeks’ gestation and race—introduce additional limitations. Reporting of ITOP data does not include the presence or absence of embryonic cardiac activity; however, embryonic cardiac activity is typically detected by 6 weeks’ and 0 days’ gestation. Weeks of gestation is reported as a whole integer, such that a measure of gestation at 6 weeks can include pregnancies from 6 weeks 0 days to 6 weeks 6 days. Thus, to simulate HB481, we used a gestational cutoff of less than 6 and 6 weeks or longer, with this cutoff representing a proxy for detection of embryonic cardiac activity. This highlights an important data issue that may arise for researchers evaluating similar laws limiting abortion to early pregnancy. Understanding the ways weeks’ gestation is reported and operationalized is critical to appropriately capture the effects of such bans, which do not ban abortion at a specific gestational cutoff but at a stage of embryonic development. Moreover, our analyses may overestimate the effect of this law if clinics and clinicians work to deliver abortion care earlier in pregnancy in response to the law.

Additionally, the ITOP data included practitioner-reported racial categories (Black, White, other, or unknown) but did not disaggregate by race and ethnicity simultaneously (ie, Hispanic, non-Hispanic Black, non-Hispanic other, non-Hispanic White). Further, as other and unknown are not racial classifications, we focused on individuals who identified as Black and White. The categorization of race in this manner erases the experiences of individuals who identify as a race or ethnicity other than Black or White, including those identifying as Alaska Native or American Indian, Asian, Native Hawaiian or other Pacific Islander, or more than 1 race. Subsequent analyses should incorporate more inclusive race data and stratify by race and ethnicity when possible. Based on the way the DPH provided the aggregate data, we were unable to examine data at multiple levels of stratification beyond weeks’ gestation and 1 sociodemographic variable, thus preventing examination of inequities by intersecting identities (eg, by race and educational level at the same time).^52,53,54^ Third, because this study analyzed abortion incidence and inequity trends unique to 1 state with a highly restrictive abortion setting, results from the study may not be generalizable to other states, particularly those in which abortion is readily accessible. However, our findings may be generalizable to other states with similar laws limiting abortion to early pregnancy. Finally, because we used multiple statistical tests, there is a potential for inflation of type I errors; thus, our findings should be viewed as exploratory.

Abortion restrictions—particularly laws limiting abortion to early pregnancy like HB481—are a threat to public health, reproductive autonomy, and well-being, and our findings have multiple implications for policy and practice. First, at the federal, state, and local levels, abortion access should be expanded and protected. Prior to and in the wake of HB481, policy makers, advocates, and community organizations have identified and championed health and social support policy initiatives that could mitigate the detrimental effects of HB481, including expanding Medicaid coverage eligibility; mandating paid parental leave; ensuring schools deliver comprehensive health education, including sexual health and consent; funding local and regional abortion funds; and protecting clinicians who provide abortions, escorts, and doulas from criminal prosecution or civil litigation. Abortion funds provide critical support to individuals seeking abortion care^37^ and should be prioritized by state and local officials. The City of Atlanta recently established a local abortion fund by pledging $300 000 to Access Reproductive Care–Southeast for patient support.^55^ To the extent possible within legal restrictions, clinics and clinicians must support and accommodate groups with the greatest barriers through flexible scheduling, sliding-scale fees, telemedicine, transportation, and escorts or doulas.

## Conclusions

This cross-sectional study found that nearly 90% of abortions that were performed in Georgia in 2016 and 2017 would no longer meet eligibility requirements for legal abortion care under HB481, Georgia’s new law prohibiting abortions after detection of embryonic cardiac activity. Relative to their counterparts, fewer abortions obtained by individuals who are Black, of younger age (<20 years), and with fewer years of education would meet the eligibility requirements for legal abortion care under HB481. These findings demonstrate the potentially detrimental and inequitable effects of restrictive abortion policies on abortion access in Georgia.

## Notes

Supplement 1. **eTable 1.** Number and Percentage of Abortions Provided in Georgia, Stratified by Weeks’ Gestation and Patient Race, Age, and Educational Level **eTable 2.** Number and Percentage of Abortions Provided in Georgia, Stratified by Weeks’ Gestation and Patient Age **eTable 3.** Number and Percentage of Abortions Provided in Georgia, Stratified by Weeks’ Gestation and Patient Educational Level **eFigure 1.** Percentage of Abortions Provided in Georgia, Stratified by Weeks’ Gestation **eFigure 2.** Percentage of Abortions Provided in Georgia <6 Weeks from Last Menstrual Period, Stratified by Patient Race **eFigure 3.** Percentage of Abortions Provided in Georgia <6 Weeks from Last Menstrual Period, Stratified by Patient Age **eFigure 4.** Percentage of Abortions Provided in Georgia <6 Weeks from Last Menstrual Period, Stratified by Patient Educational Level

Supplement 2.
Data Sharing Statement
