# Error in Conflict of Interest Disclosures

**DOI:** 10.1001/jamanetworkopen.2023.19801

**Published:** 2023-06-06

In the Original Investigation titled “Estimation of Multiyear Consequences for Abortion Access in Georgia Under a Law Limiting Abortion to Early Pregnancy,”^1^ published March 6, 2023, a conflict of interest disclosure was omitted for Dr Newton-Levinson. This article has been corrected.
