# Supplementary material for: Estimation of Multiyear Consequences for Abortion Access in Georgia Under a Law Limiting Abortion to Early Pregnancy
Source: JAMA Netw Open. 2023 Mar 6;6(3):e231598. doi: 10.1001/jamanetworkopen.2023.1598 (PMC9989903; doi:10.1001/jamanetworkopen.2023.1598)
Supplement: Supplement 1. — eTable 1. Number and Percentage of Abortions Provided in Georgia, Stratified by Weeks’ Gestation and Patient Race, Age, and Educational Level eTable 2. Number and Percentage of Abortions Provided in Georgia, Stratified by Weeks’ Gestation and Patient Age eTable 3. Number and Percentage of Abortions Provided in Georgia, Stratified by Weeks’ Gestation and Patient Educational Level eFigure 1. Percentage of Abortions Provided in Georgia, Stratified by Weeks’ Gestation eFigure 2. Percentage of Abortions Provided in Georgia <6 Weeks from Last Menstrual Period, Stratified by Patient Race eFigure 3. Percentage of Abortions Provided in Georgia <6 Weeks from Last Menstrual Period, Stratified by Patient Age eFigure 4. Percentage of Abortions Provided in Georgia <6 Weeks from Last Menstrual Period, Stratified by Patient Educational Level [file jamanetwopen-e231598-s001.pdf]

Supplementary Online Content

Redd SK, Mosley EA, Narasimhan S, et al. Estimation of multiyear consequences for abortion access in Georgia under a law limiting abortion to early pregnancy. *JAMA Netw Open*. 2023;6(3):e231598. doi:10.1001/jamanetworkopen.2023.1598

eTable 1. Number and Percentage of Abortions Provided in Georgia, Stratified by Weeks' Gestation and Patient Race, Age, and Educational Level
eTable 2. Number and Percentage of Abortions Provided in Georgia, Stratified by Weeks' Gestation and Patient Age
eTable 3. Number and Percentage of Abortions Provided in Georgia, Stratified by Weeks' Gestation and Patient Educational Level
eFigure 1. Percentage of Abortions Provided in Georgia, Stratified by Weeks' Gestation
eFigure 2. Percentage of Abortions Provided in Georgia <6 Weeks from Last Menstrual Period, Stratified by Patient Race
eFigure 3. Percentage of Abortions Provided in Georgia <6 Weeks from Last Menstrual Period, Stratified by Patient Age
eFigure 4. Percentage of Abortions Provided in Georgia <6 Weeks from Last Menstrual Period, Stratified by Patient Educational Level

This supplementary material has been provided by the authors to give readers additional information about their work.

© 2023 Redd SK et al. *JAMA Network Open*.

eTable 1. Number and Percentage of Abortions Provided in Georgia, Stratified by Weeks' Gestation and Patient Race, Age, and Educational Level

**Year**	**Race**	**Abortions <6 Weeks in GA** No. (%)	**Abortions ≥6 Weeks in GA** No. (%)	**Total Abortions in GA**	**X^2, p-value**
2007	Black	1,346 (6.9%)	18,229 (93.1%)	19,575	*9.03, 0.003*
	White	970 (7.8%)	11,519 (92.2%)	12,489	
2008	Black	885 (4.2%)	20,111 (95.8%)	20,996	*103.09, <0.001*
	White	696 (6.9%)	9,359 (93.1%)	10,055	
2009	Black	950 (4.9%)	18,353 (95.1%)	19,303	*194.11, <0.001*
	White	804 (9.3%)	7,848 (90.7%)	8,652	
2010	Black	1,139 (5.6%)	19,245 (94.4%)	20,384	*225.11, <0.001*
	White	891 (10.6%)	7,549 (89.4%)	8,440	
2011	Black	1,091 (6.1%)	16,712 (93.9%)	17,803	*141.05, <0.001*
	White	812 (10.3%)	7,037 (89.7%)	7,849	
2012	Black	1,553 (8.7%)	16,265 (91.3%)	17,818	*173.63, <0.001*
	White	1,125 (14.1%)	6,833 (85.9%)	7,958	
2013	Black	1,454 (8.0%)	16,703 (92.0%)	18,157	*189.70, <0.001*
	White	1,077 (13.5%)	6,916 (86.5%)	7,993	
2014	Black	1,228 (6.9%)	16,675 (93.1%)	17,903	*151.63, <0.001*
	White	898 (11.4%)	6,946 (88.6%)	7,844	
2015	Black	1,935 (10.5%)	16,544 (89.5%)	18,479	*33.29, <0.001*
	White	1,006 (12.9%)	6,774 (87.1%)	7,780	
2016	Black	1,920 (9.2%)	18,944 (90.8%)	20,864	*256.93, <0.001*
	White	1,266 (15.8%)	6,745 (84.2%)	8,011	
2017	Black	1,966 (10.1%)	17,457 (89.9%)	19,423	*225.62, <0.001*
	White	1,293 (16.7%)	6,460 (83.3%)	7,753	
**2007–2017 Average**	Black	1,406 (7.3%)	17,749 (92.7%)	19,155	***126.20, <0.001***
	White	985 (11.4%)	7,635 (88.6%)	8,620	
**2016–2017 Average**	Black	1,943 (9.6%)	18,201 (90.4%)	20,144	***241.91, <0.001***
	White	1,280 (16.2%)	6,603 (83.8%)	7,882	

Induced Termination of Pregnancy (ITOP) Data, 2016–2017

© 2023 Redd SK et al. *JAMA Network Open*.

eTable 2. Number and Percentage of Abortions Provided in Georgia, Stratified by Weeks' Gestation and Patient Age

Year	Age	Abortions <6 Weeks in GA No. (%)	Abortions ≥6 Weeks in GA No. (%)	Total Abortions in GA	X^2, p-value
2007	<20 years	270 (5.2%)	4,952 (94.8%)	5,222	*57.44, <0.001*
	20-29 years	1,367 (7.2%)	17,708 (92.8%)	19,075	
	30-39 years	709 (8.6%)	7,512 (91.4%)	8,221	
	≥40 years	69 (6.8%)	948 (93.2%)	1,017	
2008	<20 years	197 (3.6%)	5,267 (96.4%)	5,464	*44.64, <0.001*
	20-29 years	1,110 (5.4%)	19,358 (94.6%)	20,468	
	30-39 years	551 (6.0%)	8,571 (94.0%)	9,122	
	≥40 years	68 (6.5%)	972 (93.5%)	1,040	
2009	<20 years	205 (4.2%)	4,714 (95.8%)	4,919	*58.55, <0.001*
	20-29 years	1,285 (6.9%)	17,464 (93.1%)	18,749	
	30-39 years	604 (6.9%)	8,112 (93.1%)	8,716	
	≥40 years	91 (8.7%)	954 (91.3%)	1,045	
2010	<20 years	247 (5.1%)	4,558 (94.9%)	4,805	*43.03, <0.001*
	20-29 years	1,502 (7.6%)	18,211 (92.4%)	19,713	
	30-39 years	696 (7.5%)	8,552 (92.5%)	9,248	
	≥40 years	101 (9.3%)	988 (90.7%)	1,089	
2011	<20 years	235 (5.4%)	4,132 (94.6%)	4,367	*69.67, <0.001*
	20-29 years	1,552 (8.4%)	17,030 (91.6%)	18,582	
	30-39 years	788 (8.8%)	8,119 (91.2%)	8,907	
	≥40 years	127 (11.7%)	954 (88.3%)	1,081	
2012	<20 years	307 (8.0%)	3,546 (92.0%)	3,853	*54.38, <0.001*
	20-29 years	1,893 (10.5%)	16,065 (89.5%)	17,958	
	30-39 years	1,054 (12.2%)	7,604 (87.8%)	8,658	
	≥40 years	147 (12.6%)	1,019 (87.4%)	1,166	
2013	<20 years	243 (7.2%)	3,138 (92.8%)	3,381	*49.94, <0.001*
	20-29 years	1,711 (9.7%)	15,942 (90.3%)	17,653	
	30-39 years	933 (10.7%)	7,778 (89.3%)	8,711	
	≥40 years	149 (13.3%)	970 (86.7%)	1,119	
2014	<20 years	184 (5.9%)	2,913 (94.1%)	3,097	*77.80, <0.001*
	20-29 years	1,364 (7.8%)	16,194 (92.2%)	17,558	
	30-39 years	845 (10.0%)	7,582 (90.0%)	8,427	
	≥40 years	129 (11.6%)	980 (88.4%)	1,109	
2015	<20 years	297 (10.3%)	2,585 (89.7%)	2,882	*30.68, <0.001*
	20-29 years	1,989 (10.8%)	16,355 (89.2%)	18,344	
	30-39 years	1,129 (12.9%)	7,622 (87.1%)	8,751	
	≥40 years	144 (12.8%)	979 (87.2%)	1,123	

© 2023 Redd SK et al. *JAMA Network Open*.

2016	<20 years	285 (9.4%)	2,749 (90.6%)	3,034	*28.36, <0.001*
	20-29 years	2,166 (10.8%)	17,935 (89.2%)	20,101	
	30-39 years	1,199 (12.2%)	8,652 (87.8%)	9,851	
	≥40 years	149 (13.4%)	963 (86.6%)	1,112	
2017	<20 years	236 (8.8%)	2,457 (91.2%)	2,693	*63.96, <0.001*
	20-29 years	2,218 (11.7%)	16,670 (88.3%)	18,888	
	30-39 years	1,269 (13.3%)	8,254 (86.7%)	9,523	
	≥40 years	186 (16.5%)	944 (83.5%)	1,130	
**2007–2017 Average**	<20 years	246 (6.2%)	3,728 (93.8%)	3,974	***56.99, <0.001***
	20-29 years	1,651 (8.8%)	17,176 (91.2%)	18,826	
	30-39 years	889 (10.0%)	8,033 (90.0%)	8,921	
	≥40 years	124 (11.3%)	970 (88.7%)	1,094	
**2016–2017 Average**	<20 years	261 (9.1%)	2,603 (90.9%)	2,864	**44.29, <0.001**
	20-29 years	2,192 (11.2%)	17,303 (88.8%)	19,495	
	30-39 years	1,234 (12.7%)	8,453 (87.3%)	9,687	
	≥40 years	168 (14.9%)	954 (85.1%)	1,121	

Induced Termination of Pregnancy (ITOP) Data, 2007–2017

© 2023 Redd SK et al. *JAMA Network Open*.

eTable 3. Number and Percentage of Abortions Provided in Georgia, Stratified by Weeks' Gestation and Patient Educational Level

Year	Age	**Abortions <6 Weeks in GA** No. (%)	**Abortions ≥6 Weeks in GA** No. (%)	**Total Abortions in GA**	**X^2, p-value**
2007	Less than HS grad	354 (6.9%)	4,780 (93.1%)	5,134	*2.89, 0.24*
	HS grad / GED	844 (6.9%)	11,353 (93.1%)	12,197	
	Some college or higher	1,068 (7.4%)	13,354 (92.6%)	14,422	
2008	Less than HS grad	171 (3.8%)	4,383 (96.2%)	4,554	*75.42, <0.001*
	HS grad / GED	846 (6.9%)	11,474 (93.1%)	12,320	
	Some college or higher	708 (5.0%)	13,348 (95.0%)	14,056	
2009	Less than HS grad	218 (5.4%)	3,785 (94.6%)	4,003	*43.59, <0.001*
	HS grad / GED	922 (7.4%)	11,609 (92.6%)	12,531	
	Some college or higher	790 (5.5%)	13,519 (94.5%)	14,309	
2010	Less than HS grad	183 (3.8%)	4,663 (96.2%)	4,846	*79.55, <0.001*
	HS grad / GED	399 (4.1%)	9,217 (95.9%)	9,616	
	Some college or higher	962 (6.3%)	14,286 (93.7%)	15,248	
2011	Less than HS grad	136 (3.5%)	3,763 (96.5%)	3,899	*53.13, <0.001*
	HS grad / GED	338 (4.2%)	7,695 (95.8%)	8,033	
	Some college or higher	825 (5.9%)	13,156 (94.1%)	13,981	
2012	Less than HS grad	202 (5.9%)	3,250 (94.1%)	3,452	*74.86, <0.001*
	HS grad / GED	442 (5.9%)	6,997 (94.1%)	7,439	
	Some college or higher	1,214 (8.8%)	12,510 (91.2%)	13,724	
2013	Less than HS grad	236 (7.1%)	3,084 (92.9%)	3,320	*106.84, <0.001*
	HS grad / GED	519 (6.3%)	7,734 (93.7%)	8,253	
	Some college or higher	1,467 (10.1%)	13,086 (89.9%)	14,553	
2014	Less than HS grad	311 (7.3%)	3,928 (92.7%)	4,239	*46.83, <0.001*

© 2023 Redd SK et al. *JAMA Network Open*.

	HS grad / GED	553 (6.5%)	7,906 (93.5%)	8,459	
	Some college or higher	1,372 (9.0%)	13,919 (91.0%)	15,291	
2015	Less than HS grad	512 (11.4%)	3,973 (88.6%)	4,485	*71.53, <0.001*
	HS grad / GED	940 (9.3%)	9,134 (90.7%)	10,074	
	Some college or higher	2,104 (12.7%)	14,419 (87.3%)	16,523	
2016	Less than HS grad	412 (9.2%)	4,069 (90.8%)	4,481	*106.05, <0.001*
	HS grad / GED	1,061 (9.3%)	10,361 (90.7%)	11,422	
	Some college or higher	2,322 (12.8%)	15,847 (87.2%)	18,169	
2017	LT HS grad	372 (9.2%)	3,670 (90.8%)	4,042	*156.57, <0.001*
	HS grad / GED	1,068 (9.9%)	9,761 (90.1%)	10,829	
	Some college or higher	2,467 (14.2%)	14,867 (85.8%)	17,334	
**2007–2017 Average**	LT HS grad	282 (6.7%)	3,941 (93.3%)	4,223	***45.85, <0.001***
	HS grad / GED	721 (7.1%)	9,386 (92.9%)	10,107	
	Some college or higher	1,391 (9.1%)	13,846 (90.9%)	15,237	
**2016–2017 Average**	LT HS grad	392 (9.2%)	3,870 (90.8%)	4,262	***130.29, <0.001***
	HS grad / GED	1,065 (9.6%)	10,061 (90.4%)	11,126	
	Some college or higher	2,395 (13.5%)	15,357 (86.5%)	17,752	

GED = general education development test; HS = high school; LT = less than

Induced Termination of Pregnancy (ITOP) Data, 2007–2017

© 2023 Redd SK et al. *JAMA Network Open*.

eFigure 1. Percentage of Abortions Provided in Georgia, Stratified by Weeks' Gestation

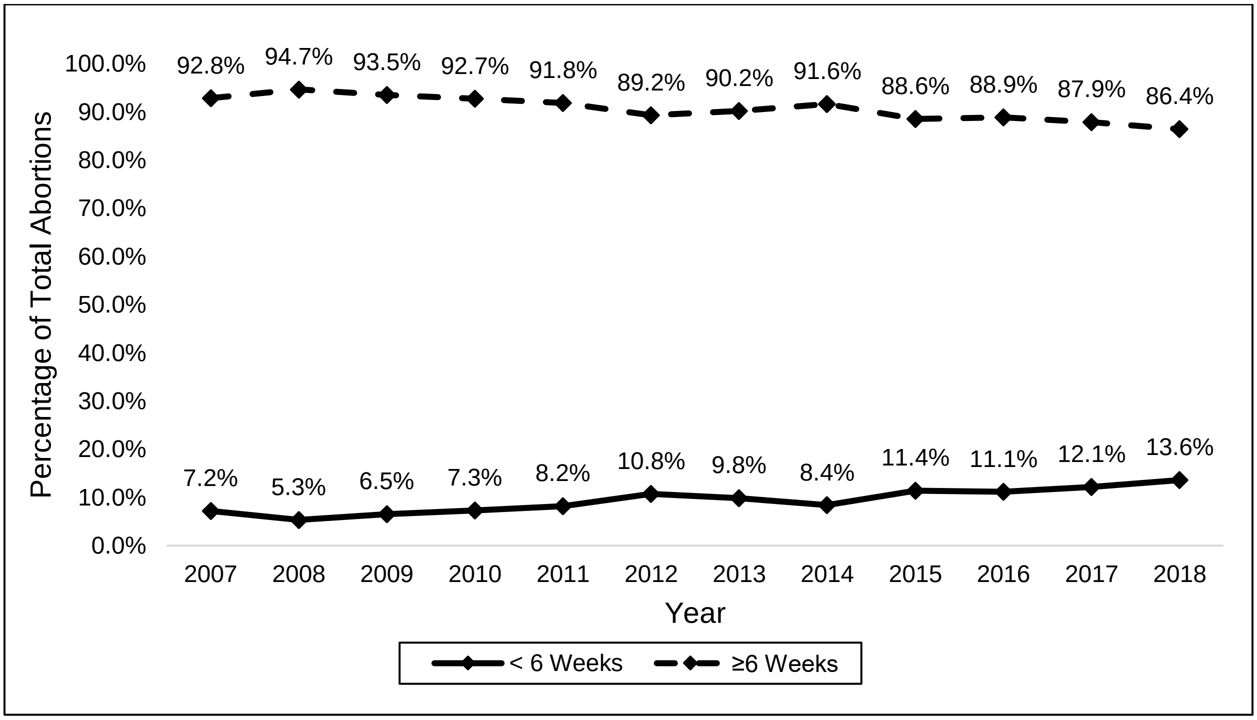

Induced Termination of Pregnancy (ITOP) Data, 2007–2017

© 2023 Redd SK et al. *JAMA Network Open*.

eFigure 2. Percentage of Abortions Provided in Georgia <6 Weeks from Last Menstrual Period, Stratified by Patient Race

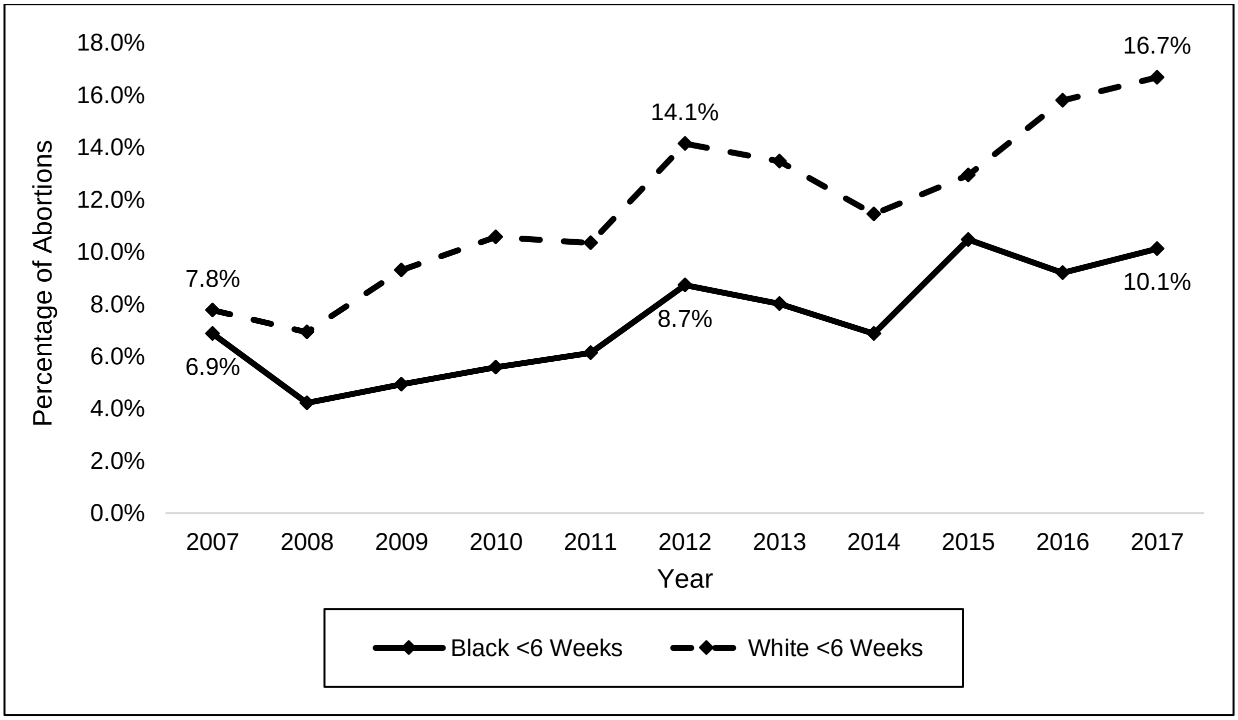

Induced Termination of Pregnancy (ITOP) Data, 2007–2017

© 2023 Redd SK et al. *JAMA Network Open*.

eFigure 3. Percentage of Abortions Provided in Georgia <6 Weeks from Last Menstrual Period, Stratified by Patient Age

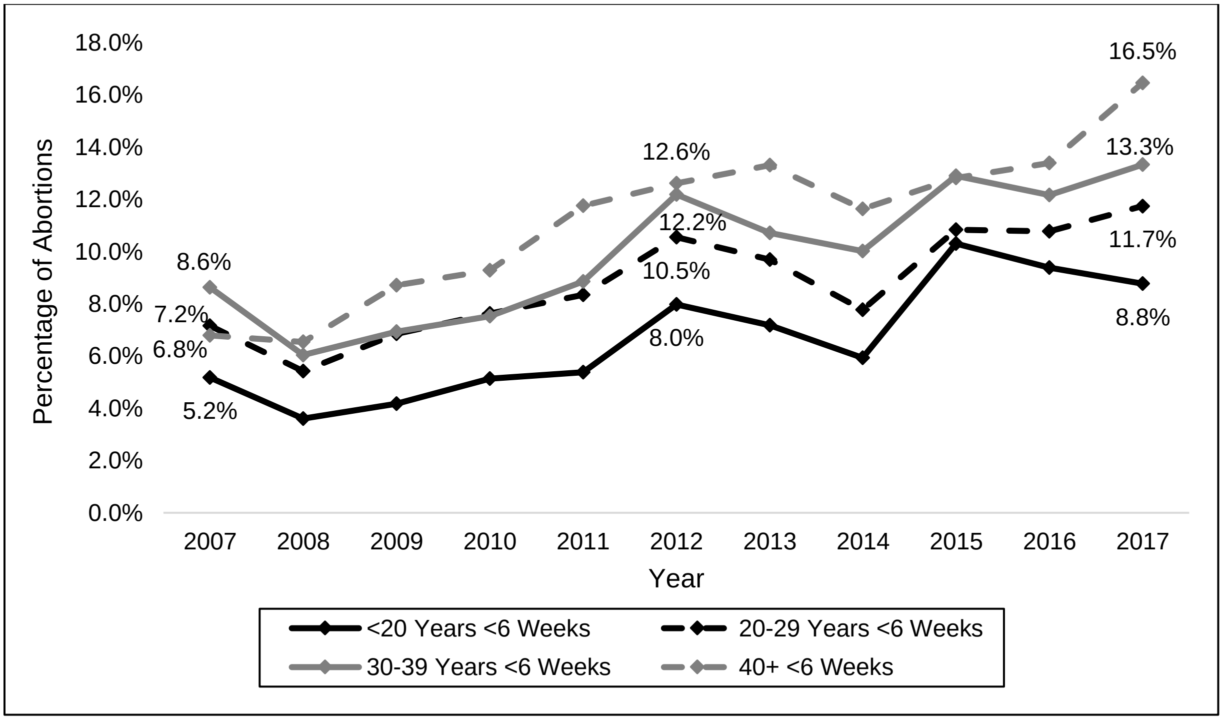

Induced Termination of Pregnancy (ITOP) Data, 2007–2017

© 2023 Redd SK et al. *JAMA Network Open*.

eFigure 4. Percentage of Abortions Provided in Georgia <6 Weeks from Last Menstrual Period, Stratified by Patient Educational Level

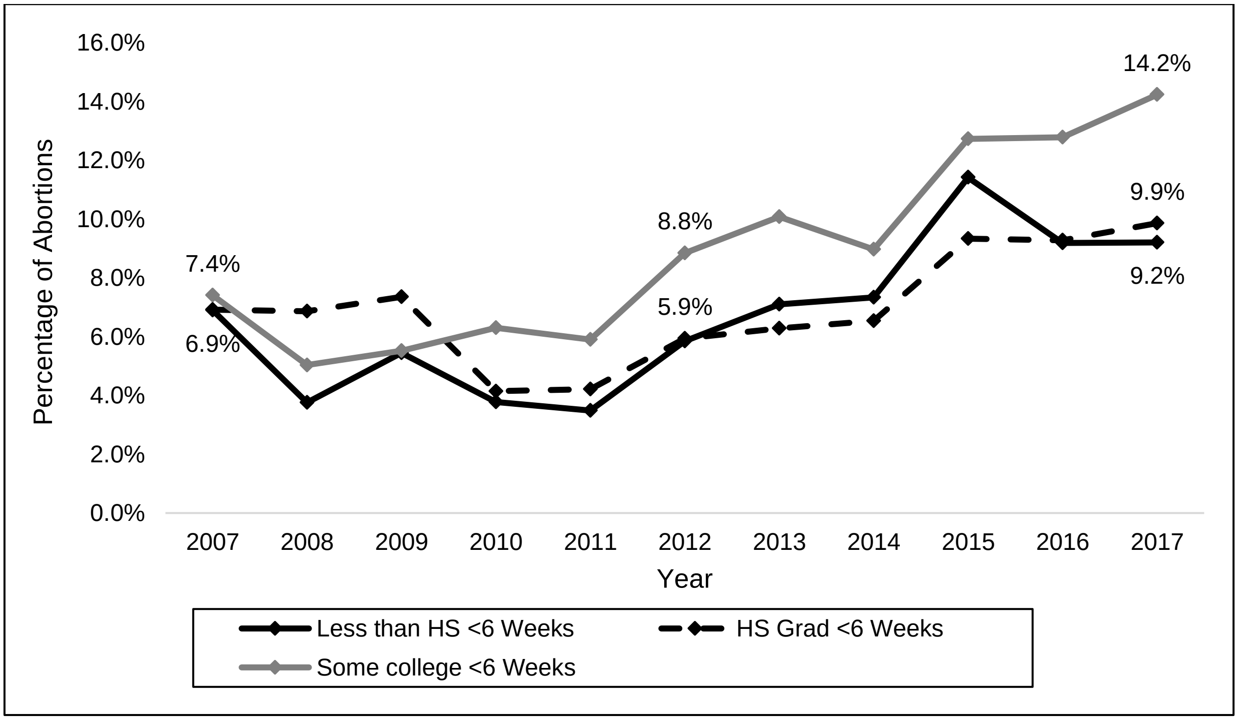

Induced Termination of Pregnancy (ITOP) Data, 2007–2017

© 2023 Redd SK et al. *JAMA Network Open*.
