# Supplementary material for: Estimation of Multiyear Consequences for Abortion Access in Georgia Under a Law Limiting Abortion to Early Pregnancy
Source: JAMA Netw Open. 2023 Mar 6;6(3):e231598. doi: 10.1001/jamanetworkopen.2023.1598 (PMC9989903; doi:10.1001/jamanetworkopen.2023.1598)
Supplement: Supplement 2. — Data Sharing Statement [file jamanetwopen-e231598-s002.pdf]

Data Sharing Statement

Redd. Estimation of Multiyear Consequences for Abortion Access in Georgia Under a Law Limiting Abortion to Early Pregnancy. *JAMA Netw Open*. Published March 06, 2023. doi:10.1001/jamanetworkopen.2023.1598

Data
Data available: No

Additional Information
Explanation for why data not available: This study used data made available via a data request to the Georgia Department of Public Health that the author does not have permission to share. Requests to access the data should be directed to the Georgia Department of Public Health.
